# Supplementary material for: Integrating apaQTL and eQTL analysis identifies a potential causal variant associated with lung adenocarcinoma risk in the Chinese population
Source: Commun Biol. 2024 Jul 13;7:860. doi: 10.1038/s42003-024-06502-0 (PMC11246497; doi:10.1038/s42003-024-06502-0)
Supplement: Supplementary file 2 — Supplementary Information [file 42003_2024_6502_MOESM2_ESM.pdf]

**Supplementary Information**

# Integrating apaQTL and eQTL analysis identifies a potential causal variant associated with lung adenocarcinoma risk in the Chinese population

Huiwen Xu[1*], Yutong Wu[1*], Qiong Chen[1*], Qianyao Meng[2], Na Qin[3, 4], Wendi Zhang[1], Xiaobo Tao[1], Siqi Li[1], Tian Tian[1], Lei Zhang[1], Hongxia Ma[3, 4], Jiahua Cui[1#], Minjie Chu[1#]

This file contains:
• - Supplementary Figure1: The gating strategies of flow cytometry in this study
• - Supplementary Figure2: Uncropped western blots related to Figure 4a,4c
• - Supplementary Figure3: Uncropped gel images related to Figure 6b
• - Supplementary Table 1: Antibodies used in this study
• - Supplementary Table 2: Oligomer sequences for cell transfection
• - Supplementary Table 3: Oligomer sequences for cell transfection
Oligomer sequence for rapid amplification of 3′- cDNA ends (3′RACE) experiment and qRT-PCR Assay

**Supplementary Figure 1: The gating strategies of flow cytometry in this study**

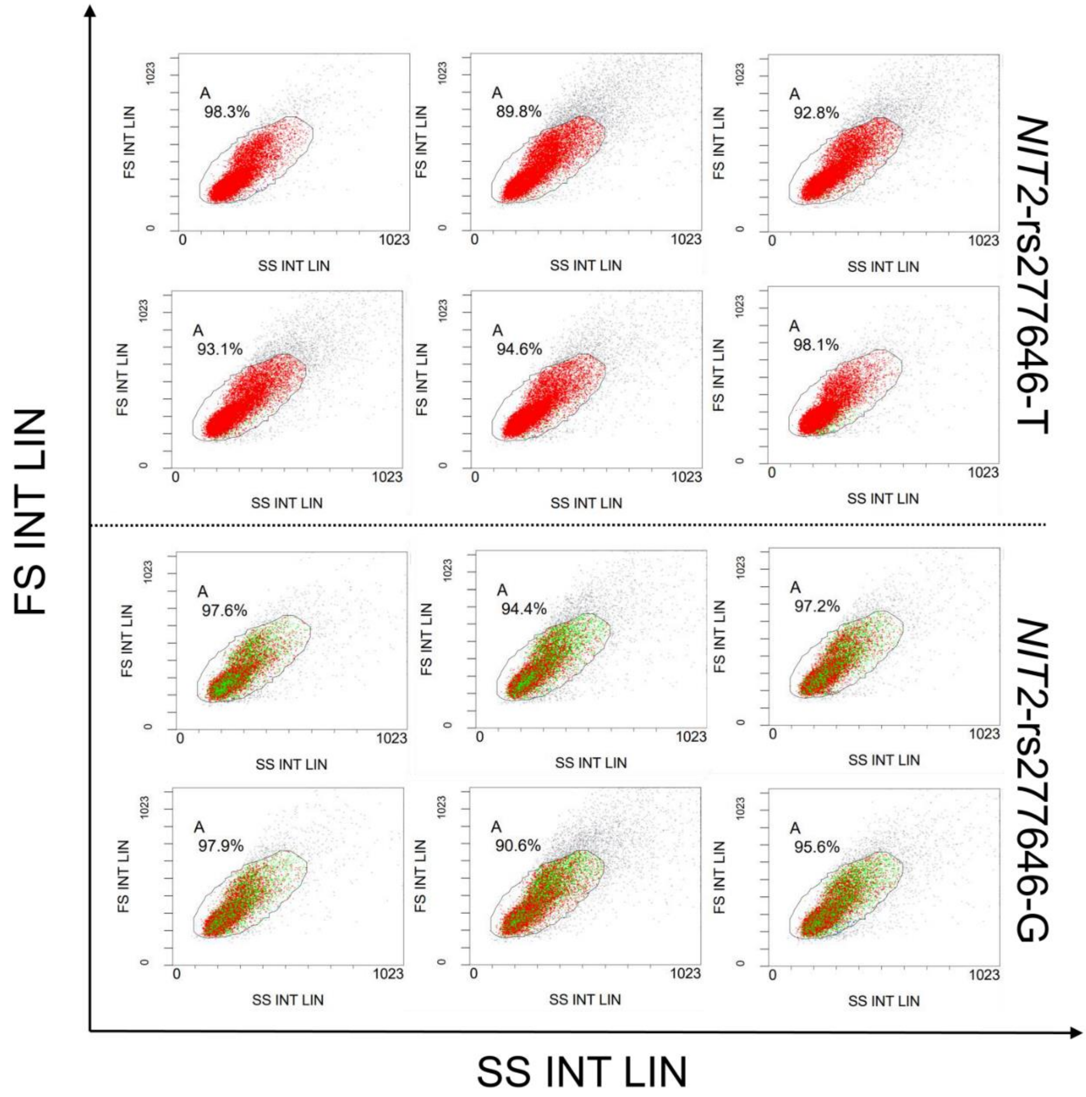

## Supplementary Figure 2: Uncropped western blots related to Figure 4a,4c

**Figure 4a. The expression of NIT2-rs277646-T and NIT2-rs277646-G**

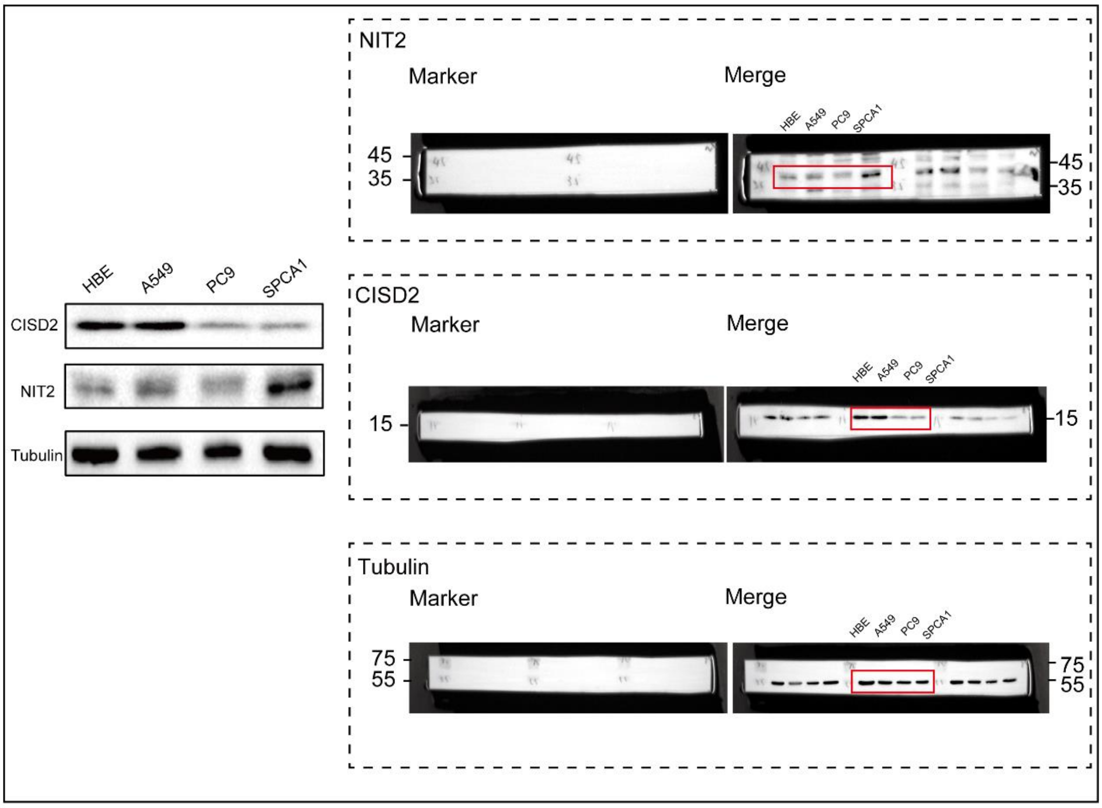


**Figure 4c. The expression of NIT2-rs277646-T and NIT2-rs277646-G**

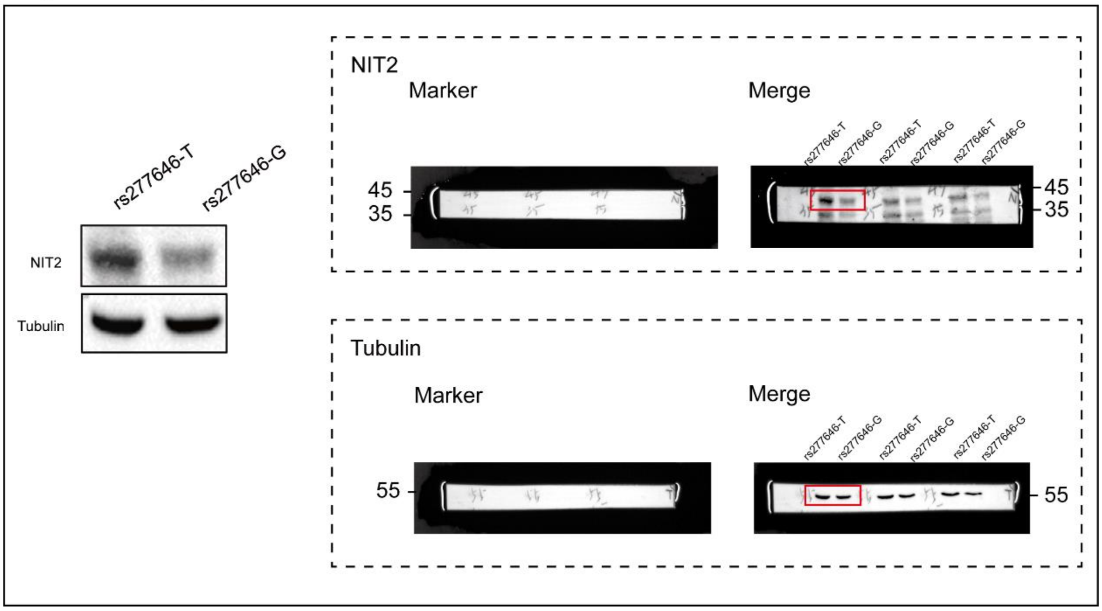

## Supplementary Figure 3: related to Figure 6b. Uncropped gel images

**Figure 4b. Agarose gel map of PCR products of 3′RACE under different alleles of rs277646**

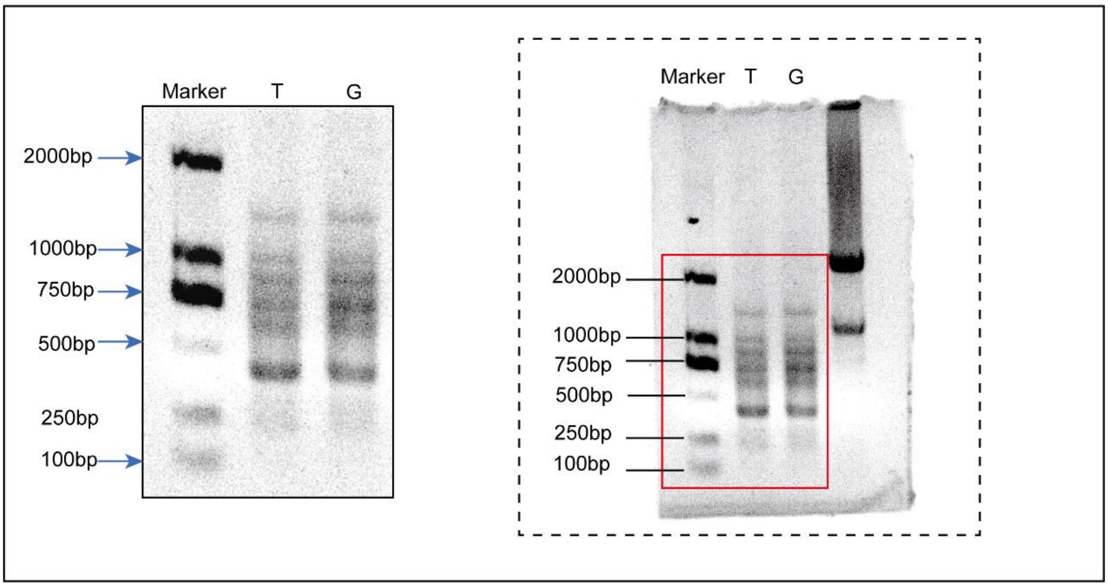

**Supplementary Table 1: Antibodies used in this study**

| Antibody (clone) | Manufacturer (Catalog number) | Usage: Dilution |
|---|---|---|
| anit-NIT2 | Proteintech (68169-1-lg) | WB: 1:5000 |
| anti-CISD2 | Proteintech (66082-1-lg) | WB: 1:2000 |
| Tubulin | Beyotime (AT819) | WB: 1:1000 |
| ki67 | Servicebio (GB111141-100) | IHC: 1: 500 |
| cleaved caspase 3 | CST (9664S) | IHC: 1: 1000 |

WB: western blot; IHC: immunohistochemistry

**Supplementary Table 2: Oligomer sequences for cell transfection**

| Name | Sequence |
|---|---|
| *NIT2*-KOF | caccGTATTTCAGCCAGCTTCTTC |
| *NIT2*-KOR | aaacGAAGAAGCTGGCTGAAATAC |

**Supplementary Table 3: Oligomer sequences for cell transfection**
**Oligomer sequence for rapid amplification of 3′- cDNA ends (3′RACE) experiment and qRT-PCR Assay**

| Name | Sequence |
|---|---|
| *NIT2*-GSP-F1 | ATCTACGCACAGGGGGGTCCAG |
| *NIT2*-GSP-F2 | CTCATTATGCTGACATTCCACG |
| Homo-*NIT2*-3′UTR-Long-F | CTGTGGAGATGAAAAAGCCCTAAAG |
| Homo-*NIT2*-3′UTR-Long-R | CGTGGAAAATGTCAGCATAATGAG |
| Homo-*NIT2*-3′UTR-Short-F | ATCTACGCACAGAGAGGCTGCCAG |
| Homo-*NIT2*-3′UTR-Short-R | AGAACCTCCCCCCAAGGGTTCAC |
| GAPDH-F | TGTTGCCATCAATGACCCCTT |
| GAPDH-R | CTCCACGACGTACTCAGCG |
