# Supplementary material for: Integrating apaQTL and eQTL analysis identifies a potential causal variant associated with lung adenocarcinoma risk in the Chinese population
Source: Commun Biol. 2024 Jul 13;7:860. doi: 10.1038/s42003-024-06502-0 (PMC11246497; doi:10.1038/s42003-024-06502-0)
Supplement: Supplementary file 3 — Description of additional supplementary files [file 42003_2024_6502_MOESM3_ESM.pdf]

Description of Additional Supplementary Files

File name: Supplementary Data 1
Description: The source behind the graphs in the paper.

File name: Supplementary Data 2
Description: Source data of tumor growth related to Figure 4g.

File name: Supplementary Software
Description: The code used in this study.
