# Supplementary material for: Integrating apaQTL and eQTL analysis identifies a potential causal variant associated with lung adenocarcinoma risk in the Chinese population
Source: Commun Biol. 2024 Jul 13;7:860. doi: 10.1038/s42003-024-06502-0 (PMC11246497; doi:10.1038/s42003-024-06502-0)
Supplement: Supplementary file 6 — Supplementary Software [file 42003_2024_6502_MOESM6_ESM.pdf]

```
# independent Student′s t test and paired t test
install.packages("psych") # Install the package "psych"
library(psych) # Call the package "psych"
describe(mydata$LUAD) # The concentration and dispersion trends of LUAD are
described
describe(mydata$Normal) # The concentration and dispersion trends of Normal group
are described
t.test(mydata$LUAD,mydata$Normal,paired = TRUE) # Paired sample t test
t_test <- t.test(expression of gene~Group, my data, paired = FALSE, alternative =
'two.sided') # independent Student′s t test

# Spearman rank correlation
corr <- cor. test (NIT2, PDUI, method = ' spearman ')
corr <- cor. test (CISD2, PDUI, method = ' spearman ')

# Logistic
data$y<-factor(data$y,levels=c(0,1),labels=c("Normal","LUAD"))# Binary variables are
treated as factor
summary(data)
model<-glm(y~.,data=a,family=binomial())#Incorporate variables into the model
summary(model)
exp(cbind("OR"=coef(mode l),confint(model)))# Calculate the OR value

#Visualization
library(ggplot2)
library(ggsignif)
library(ggpubr)
library(tidyverse)
library(gghalves)
library(dplyr)

ggplot(data,aes(x=Group,y=NIT2,fill=Group))+
geom_bar(stat="summary",fun=mean)+
stat_summary(fun.data = 'mean_sd', geom = "errorbar", position = position_dodge( .9))+
geom_jitter(data=data, mapping=aes(x=Group,y=NIT2))+
   geom_signif(comparisons = list(c("T allele", "G allele")),
                      annotation=c("**"),
                      test = "t.test")

ggplot(data,aes(x=Group,y=NIT2,color=Group))+
stat_boxplot(geom="errorbar", position=position_dodge(0.75))+
geom_boxplot(position=position_dodge(0.75))+
geom_jitter(position = position_jitterdodge(jitter.width =0.25, jitter.height = 0.0, dodge.width
= 0.75))
```

```
ggplot(data , aes(x = Group, y = NIT2, fill = Group))+
geom_half_violin(data = iris_LUAD, side = 'r', position = position_nudge(x = .20, y = 0))+
geom_half_violin(data = iris_Normal, side = 'l', position = position_nudge(x = -0.20, y = 0))
```
