# Supplementary material for: Integrating apaQTL and eQTL analysis identifies a potential causal variant associated with lung adenocarcinoma risk in the Chinese population
Source: Commun Biol. 2024 Jul 13;7:860. doi: 10.1038/s42003-024-06502-0 (PMC11246497; doi:10.1038/s42003-024-06502-0)
Supplement: Supplementary file 7 — Reporting Summary [file 42003_2024_6502_MOESM7_ESM.pdf]

nature portfolio

Corresponding author(s): Minjie Chu
Last updated by author(s): 2024.06.04

Reporting Summary

Nature Portfolio wishes to improve the reproducibility of the work that we publish. This form provides structure for consistency and transparency in reporting. For further information on Nature Portfolio policies, see our Editorial Policies and the Editorial Policy Checklist.

Please do not complete any field with "not applicable" or n/a. Refer to the help text for what text to use if an item is not relevant to your study.
For final submission: please carefully check your responses for accuracy; you will not be able to make changes later.

Statistics

For all statistical analyses, confirm that the following items are present in the figure legend, table legend, main text, or Methods section.

n/a	Confirmed	
☐	☒	The exact sample size (n) for each experimental group/condition, given as a discrete number and unit of measurement
☐	☒	A statement on whether measurements were taken from distinct samples or whether the same sample was measured repeatedly
☐	☒	The statistical test(s) used AND whether they are one- or two-sided<br>*Only common tests should be described solely by name; describe more complex techniques in the Methods section.*
☐	☒	A description of all covariates tested
☒	☐	A description of any assumptions or corrections, such as tests of normality and adjustment for multiple comparisons
☐	☒	A full description of the statistical parameters including central tendency (e.g. means) or other basic estimates (e.g. regression coefficient) AND variation (e.g. standard deviation) or associated estimates of uncertainty (e.g. confidence intervals)
☐	☒	For null hypothesis testing, the test statistic (e.g. F, t, r) with confidence intervals, effect sizes, degrees of freedom and P value noted<br>*Give P values as exact values whenever suitable.*
☒	☐	For Bayesian analysis, information on the choice of priors and Markov chain Monte Carlo settings
☒	☐	For hierarchical and complex designs, identification of the appropriate level for tests and full reporting of outcomes
☐	☒	Estimates of effect sizes (e.g. Cohen's d, Pearson's r), indicating how they were calculated

Our web collection on statistics for biologists contains articles on many of the points above.

Software and code

Policy information about availability of computer code

Data collection	For immunohistochemistry (IHC) analysis: EVOSTM M7000 Imaging System (Thermo Fisher Scientific, USA)<br>For Flow cytometry for apoptosis analysis: FACScan flow cytometer (Becton, CA)
Data analysis	All statistical analyses were performed by R version 4.1.1 software. Part of the data processing software is done by imageJ.

For manuscripts utilizing custom algorithms or software that are central to the research but not yet described in published literature, software must be made available to editors and reviewers. We strongly encourage code deposition in a community repository (e.g. GitHub). See the Nature Portfolio guidelines for submitting code & software for further information.

Data

Policy information about availability of data

All manuscripts must include a data availability statement. This statement should provide the following information, where applicable:
- Accession codes, unique identifiers, or web links for publicly available datasets
- A description of any restrictions on data availability
- For clinical datasets or third party data, please ensure that the statement adheres to our policy

Public databases:
TCGA-LUAD gene expression data and clinical information: [https://portal.gdc.cancer.gov/]
transcriptomics database of LUAD (49 paired LUAD tumor tissue and adjacent non-tumor tissues) from the Chinese population: GSE140343 Proteomics data of the Chinese LUAD cohort: iProx: IPX0001804000
LUAD genes related to APA events: [https://wlcb.oit.uci.edu/3aQTLatlas]

Research involving human participants, their data, or biological material

Policy information about studies with human participants or human data. See also policy information about sex, gender (identity/presentation), and sexual orientation and race, ethnicity and racism.

Reporting on sex and gender	Gender stratification was not involved in the study. Gender information for the sample was not available
Reporting on race, ethnicity, or other socially relevant groupings	China
Population characteristics	Patients with LUAD and healthy controls
Recruitment	All participants in the present study signed an informed consent form
Ethics oversight	Approved by local internal review committee or ethics committee.

Note that full information on the approval of the study protocol must also be provided in the manuscript.

Field-specific reporting

Please select the one below that is the best fit for your research. If you are not sure, read the appropriate sections before making your selection.

☒ Life sciences ☐ Behavioural & social sciences ☐ Ecological, evolutionary & environmental sciences

For a reference copy of the document with all sections, see nature.com/documents/nr-reporting-summary-flat.pdf

Life sciences study design

All studies must disclose on these points even when the disclosure is negative.

Sample size	A total of 8,762 LUAD cases and 13,328 healthy controls were included in the susceptibility study.
Data exclusions	Briefly, Dai et al. first excluded samples with genotype completion rates <95%, gender discrepancies, familial relationships, extreme heterozygosity rates (6 S.D. from the mean), or population stratification (>6 S.D. from the mean on any one of the top ten principal components). Then, Dai et al. excluded duplicate markers or SNPs with call rates <95%, minor allele frequencies (MAFs) <0.01 or Hardy-Weinberg equilibrium (HWE) P value <1×10−7 in controls or HWE P value <1×10−12 in cases. Finally, we select the LUAD sample and its SNP information
Replication	The findings made here have not been replicated in another dataset.
Randomization	The study is descriptive, thus the randomization was not done.
Blinding	Genetic testing was done with lab staff blinded about the case-control status of the samples, as well as the other characteristics of the samples.

Reporting for specific materials, systems and methods

We require information from authors about some types of materials, experimental systems and methods used in many studies. Here, indicate whether each material, system or method listed is relevant to your study. If you are not sure if a list item applies to your research, read the appropriate section before selecting a response.

Materials & experimental systems

n/a	Involved in the study	
☐	☒	Antibodies
☐	☒	Eukaryotic cell lines
☒	☐	Palaeontology and archaeology
☐	☒	Animals and other organisms
☒	☐	Clinical data
☒	☐	Dual use research of concern
☒	☐	Plants

Methods

n/a	Involved in the study	
☒	☐	ChIP-seq
☐	☒	Flow cytometry
☒	☐	MRI-based neuroimaging

Antibodies

Antibodies used	Western blot analysis was conducted and the antibodies we used were anit-NIT2 (1:5000, Proteintech), anti-CISD2 (1:2000, Proteintech) and Tubulin (1:1000, Beyotime). The following antibodies were used for IHC: ki67 (GB111141-100, Servicebio, Shanghai, China) and cleaved caspase 3 (9664S, CST, USA)
Validation	All antibodies have been validated by their manufacturers; the manufacturer's website es stating the validation (including the species and application) of each antibody were listed above.

Eukaryotic cell lines

Policy information about cell lines and Sex and Gender in Research

Cell line source(s)	The LUAD cell lines used in this study were A549, PC9 and SPCA1, as well as HBE (human bronchial epithelial cell line), all cell lines were purchased from American Type Culture Collection (ATCC).
Authentication	!pnфΣ t/фΣ {t/!ʍ,l .9 and HEK 293T cell
Mycoplasma contamination	All cell lines were tested negative for mycoplasma contamination.
Commonly misidentified lines (See ICLAC register)	bƻ ŏƻYΥƻyƒè YƛăƛŘŜyΎüƛŦƛŜŘ ŏŜff fƛyŜă øŜ₩Ŝ dzăŜŘ ƛyΎüKŜ ăúdzŘè

Animals and other research organisms

Policy information about studies involving animals; ARRIVE guidelines recommended for reporting animal research, and Sex and Gender in Research

Laboratory animals	Species: Balb/c-nu Grade: SPF
Wild animals	We did not use wild animals.
Reporting on sex	Male
Field-collected samples	12 samples
Ethics oversight	The animal studies were approved by the Animal Ethics Committee of Nantong University, China (S20220224-006).

Flow Cytometry

Plots

Confirm that:

- [x] The axis labels state the marker and fluorochrome used (e.g. CD4-FITC).
- [x] The axis scales are clearly visible. Include numbers along axes only for bottom left plot of group (a 'group' is an analysis of identical markers).
- [x] All plots are contour plots with outliers or pseudocolor plots.
- [x] A numerical value for number of cells or percentage (with statistics) is provided.

Methodology

Sample preparation	Cells were harvested into 5 ml centrifuge tubes at 37°C and washed with PBS.
Instrument	After adding 195 µL Annexin-V-FITC conjugate, 5 µL Annexin-V-FITC and 10 µL PI stain, the cells were incubated at room temperature for 15 minutes in the dark.
Software	FACScan flow cytometer (Becton, CA) equipped with CellQuest Software (Becton Dickinson).
Cell population abundance	For any sorting experiment, the cells are then counted manually to determine the number of cells obtained (approximately 1000); The purity of the post-sorting population is determined by flow cytometric analysis after any initial sorting.
Gating strategy	By setting specific gating conditions, cells stained by PI can be effectively screened.

- [ ] Tick this box to confirm that a figure exemplifying the gating strategy is provided in the Supplementary Information.

This checklist template is licensed under a Creative Commons Attribution 4.0 International License, which permits use, sharing, adaptation, distribution and reproduction in any medium or format, as long as you give appropriate credit to the original author(s) and the source, provide a link to the Creative Commons license, and indicate if changes were made. The images or other third party material in this article are included in the article's Creative Commons license, unless indicated otherwise in a credit line to the material. If material is not included in the article's Creative Commons license and your intended use is not permitted by statutory regulation or exceeds the permitted use, you will need to obtain permission directly from the copyright holder. To view a copy of this license, visit http://creativecommons.org/licenses/by/4.0/
